# Electronegative low density lipoprotein induces renal apoptosis and fibrosis: STRA6 signaling involved[S]

**DOI:** 10.1194/jlr.M067215

**Published:** 2016-08

**Authors:** Chao-Hung Chen, Liang-Yin Ke, Hua-Chen Chan, An-Sheng Lee, Kun-Der Lin, Chih-Sheng Chu, Mei-Yueh Lee, Pi-Jung Hsiao, Chin Hsu, Chu-Huang Chen, Shyi-Jang Shin

**Affiliations:** Graduate Institute of Medicine,* College of Medicine, Kaohsiung Medical University, Kaohsiung, Taiwan; Department of Medical Laboratory Science and Biotechnology, College of Health Sciences,† College of Medicine, Kaohsiung Medical University, Kaohsiung, Taiwan; Lipid Science and Aging Research Center,§ College of Medicine, Kaohsiung Medical University, Kaohsiung, Taiwan; Departments of Internal Medicine *** College of Medicine, Kaohsiung Medical University, Kaohsiung, Taiwan; Physiology,††† College of Medicine, Kaohsiung Medical University, Kaohsiung, Taiwan; Department of Medicine,** Mackay Medical College, New Taipei, Taiwan; Divisions of Endocrinology and Metabolism †† Department of Internal Medicine, Kaohsiung Medical University Hospital, Kaohsiung, Taiwan; Cardiology,§§ Department of Internal Medicine, Kaohsiung Medical University Hospital, Kaohsiung, Taiwan; Department of Vascular and Medical Research,§§§ Texas Heart Institute, Houston, TX

**Keywords:** lectin-like oxidized LDL receptor-1, stimulated by retinoic acid 6, renal tubular cells

## Abstract

Dyslipidemia has been proven to capably develop and aggravate chronic kidney disease. We also report that electronegative LDL (L5) is the most atherogenic LDL. On the other hand, retinoic acid (RA) and RA receptor (RAR) agonist are reported to be beneficial in some kidney diseases. “Stimulated by retinoic acid 6” (STRA6), one retinol-binding protein 4 receptor, was recently identified to regulate retinoid homeostasis. Here, we observed that L5 suppressed STRA6 cascades [STRA6, cellular retinol-binding protein 1 (CRBP1), RARs, retinoid X receptor α, and retinol, RA], but L5 simultaneously induced apoptosis and fibrosis (TGFβ_1_, Smad2, collagen 1, hydroxyproline, and trichrome) in kidneys of L5-injected mice and L5-treated renal tubular cells. These L5-induced changes of STRA6 cascades, renal apoptosis, and fibrosis were reversed in kidneys of LOX1^−/−^ mice. LOX1 RNA silencing and inhibitor of c-Jun N-terminal kinase and p38MAPK rescued the suppression of STRA6 cascades and apoptosis and fibrosis in L5-treated renal tubular cells. Furthermore, *crbp1* gene transfection reversed downregulation of STRA6 cascades, apoptosis, and fibrosis in L5-treated renal tubular cells. For mimicking STRA6 deficiency, efficient silencing of STRA6 RNA was performed and was found to repress STRA6 cascades and caused apoptosis and fibrosis in L1-treated renal tubular cells. In summary, this study reveals that electronegative L5 can cause kidney apoptosis and fibrosis via the suppression of STRA6 cascades, and implicates that STRA6 signaling may be involved in dyslipidemia-mediated kidney disease.

Previous epidemiology studies have demonstrated that dyslipidemia is an important risk factor for the development and progression of chronic kidney disease (1–5). Furthermore, it has been shown that lowering cholesterol concentration with statins might reduce the rate of renal function decline in several clinical trials (6–8). However, mechanisms of lipid-mediated kidney injury have not been well investigated because oxidized LDL (oxLDL) produced by oxidizing LDL with CuSO_4_ was used in most of these studies (9–14). On the other hand, we have reported that human plasma LDL can be chromatographically divided into five subfractions (L1–L5) with increasing electronegativity. By using native electronegative LDL, we have demonstrated that the most electronegative subfraction, L5, is the only one capable of inducing endothelial cell apoptosis (15, 16), platelet aggregation (17), C-reactive protein overproduction (18), and impairment of endothelial cell regeneration (19, 20). Other investigators have also reported that electronegative LDL can promote TG accumulation in cardiomyocytes (21) and induce inflammasome activation in human macrophages (22). Plasma L5 levels are elevated in patients with high cardiovascular risks, such as hypercholesterolemia and type 2 diabetes (15, 20). Recently, LDL from patients with stage 2 chronic kidney disease was reported to be more electronegative than LDL from control subjects (23). Therefore, it is worthy of investigating whether L5 can cause kidney damage.

Vitamin A (retinol) and its derivatives regulate a wide range of crucial biological functions, such as development, differentiation, metabolism, and immunity. Circulating retinol is bound to retinol-binding protein 4 (RBP4) and connects with transthyretin to produce a retinol-RBP4-transthyretin complex (24, 25). This complex is recognized by one RBP4 receptor, termed “stimulated by retinoic acid 6” (STRA6), which transports retinol into cells from the retinol complex (26, 27). The STRA6-mediated translocation of retinol requires the participation of cellular retinol-binding protein 1 (CRBP1), an intracellular retinol acceptor, as well as retinol-metabolizing enzymes, such as lecithin:retinol acyltransferase (28–30). Retinol in cells is thereafter metabolized into retinoic acid (RA). RA exerts its function by binding to cytosolic nuclear receptors, including RA receptors (RARs) and retinoid X receptor (RXR), which can activate the transcription of numerous target genes (24, 25). RA has also been reported to capably inhibit inflammation, apoptosis, and proliferation (31, 32). Furthermore, RA and RARα agonist have been reported to have beneficial effects in several experimental models of kidney diseases (33–36). Accordingly, it is reasonable to hypothesize that STRA6 and its cascades could be altered and involved in pathogenesis of kidney diseases. Therefore, the main goals of this study were to explore: *1*) whether L5 treatment can cause kidney apoptosis and fibrosis in L5-treated mice and renal tubule cells; *2*) whether L5 treatment can simultaneously alter STRA6, CRBP1, retinol, RA, RARs, and RXRα and *3*) whether the alteration of STRA6-mediated cascades are involved in kidney apoptosis and fibrosis caused by L5.

## MATERIALS AND METHODS

### Materials

Primary antibodies for Western blot analysis were as follows: anti-LOX1 antibody (Santa Cruz Biotechnology, Santa Cruz, CA), anti-STRA6 antibody (ABGENT, San Diego, CA), anti-CRBP1 antibody (Santa Cruz Biotechnology), anti-RARα antibody (Santa Cruz Biotechnology), anti-RARγ antibody (Santa Cruz Biotechnology), anti-RXRα antibody (Santa Cruz Biotechnology), anti-c-Jun N-terminal kinase (JNK) antibody (Santa Cruz Biotechnology), anti-pJNK antibody (Abcam, Cambridge, MA), anti-p38MAPK antibody (ABGENT), anti-p-p38MAPK antibody (ABGENT), anti-pSmad2 antibody (Santa Cruz Biotechnology), anti-Smad2 antibody (Santa Cruz Biotechnology), anti-TGFβ_1_ (Santa Cruz Biotechnology), anti-caspase 3 antibody (Santa Cruz Biotechnology), anti-collagen 1 antibody (Santa Cruz Biotechnology), and anti-actin antibody (Millipore, Temecula, CA). Secondary antibodies for Western blot analysis as HRP-conjugate antibody were purchased from Millipore. The inhibitors of JNK, SP600125 and p38MAPK, SB203580 were purchased from Sigma-Aldrich (St. Louis, MO).

### L5 of human subjects

The treatment of all human subjects in this study was performed in accordance with the ethical guidelines of the 1975 Declaration of Helsinki and approved by the Kaohsiung Medical University Hospital Institutional Review Board (KMUH-IRB-20130397). Plasma L1 and L5 were obtained from blood samples of 30 patients with metabolic syndrome (MetS) in Kaohsiung Medical University Hospital, and used for animal and cell experimentation. MetS was defined according to modification criteria for Asian populations from NCEP in Adults (ATP-III). Subjects with more than three of the following five criteria were diagnosed as MetS: *1*) waist circumference ≥90 cm in men and ≥80 cm in women; *2*) TG ≥150 mg/dl; *3*) HDL cholesterol <40 mg/dl in men and <50 mg/dl in women; *4*) blood pressure >130/85 mmHg or taking antihypertensive medication; and *5*) fasting plasma glucose ≥100 mg/dl and/or taking antidiabetic agents. Human blood samples were added into 50 mU/ml aprotinin, 1% ampicillin/streptomycin, and 5 mmol/l EDTA immediately after collection to prevent contamination and experimental oxidation. LDL particles were isolated by sequential potassium bromide density centrifugation to remove chylomicron, VLDL, and IDL fractions, and yielded LDL at a final density of 1.019–1.063. LDL was equilibrated by dialysis in a column loaded with 20 mmol/l Tris-HCl (pH 8.0), 0.6 mmol EDTA, and 0.01% NaN_3_. LDL samples were injected into a UnoQ12 anion-exchange column by using anion-exchange columns (Uno-Q12; Bio-Rad Laboratories, Inc.) with the AKTA fast protein liquid chromatography system (GE Healthcare Life Sciences, Little Chalfont, UK) and eluted with multistep chloride gradient and then divided into subfractions L1 and L5. The proportion of L5 levels was 5.3 ± 6.9% in MetS subjects. The value was significantly higher than that (2.1 ± 1.4%) in healthy subjects.

### Animal studies

Eight-week-old chow diet-fed C57B6/J male mice (n = 3) were injected with 150 μl L5 or L1 (1 mg/kg) through the tail vein every day for 4 weeks. An equal volume of saline was also used as control. Eight-week-old chow diet-fed LOX1 knockout mice (LOX1^−/−^, n = 3) were injected with 150 μl L5 (1 mg/kg) daily for 4 weeks. At the end of the experiments, animals were euthanized with chloral hydrate by ip injection, and kidneys were harvested for further analyses. The Institutional Animal Care and Use Committee of Kaohsiung Medical University approved all animal experiments (IACUC number 102149). C57B6/J mice were purchased from BioLASCO Taiwan Co., Ltd. (Taipei, Taiwan). All animals were housed and cared for in a pathogen-free facility at Kaohsiung Medical University.

### Cell culture

Human renal proximal tubular epithelial cells (HK-2; ATCC number CRL-2190) were cultured in keratinocyte serum-free medium (Invitrogen, Carlsbad, CA) containing 5 ng/ml recombinant epidermal growth factor, 40 μg/ml bovine pituitary extract supplemented with 100 U/ml penicillin (Invitrogen), 100 mg/ml streptomycin (Invitrogen) at 37°C under a 95% air and 5% CO_2_ condition. Primary mouse renal tubular epithelial cells (RTECs) were grown in DMEM/F-12 culture medium (Invitrogen) containing 10% fetal bovine serum, 0.01 μg/ml recombinant epidermal growth factor, transferrin (5 μg/ml), insulin (5 μg/ml), hydrocortisone (50 μM), 100 U/ml of penicillin, and 100 μg/ml of streptomycin in a humidified atmosphere of 95% air and 5% CO_2_ at 37°C. RTECs were identified by cell morphology and tested for mycoplasma contamination. RTECs were stimulated with native human L1 (50 μg/ml) or L5 (50 μg/ml) for 24 h with or without 10 μM SP600125 or 10 μM SB203580 in experiments.

### siRNA transfection

For cell experiments of LOX1 and STRA6 knockdown, HK-2 cells were seeded in 6-well plates at a density of 2 × 10^5^ cells per well in 2 ml antibiotic-free medium and then cells were cultured at 37°C and 5% CO_2_ until the cell growth covered 80% of the area of the dish. After overnight incubation, negative control scramble siRNA (Santa Cruz Biotechnology Inc.), LOX1 siRNA (Santa Cruz Biotechnology Inc.) or STRA6 siRNA (Santa Cruz Biotechnology Inc.) was mixed into transfection reagent (Santa Cruz Biotechnology Inc.). The transfection medium (Santa Cruz Biotechnology Inc.), and the mixture were added into cells and incubated for 7 h. These cells were placed in fresh medium for 24 h, and then stimulated with PBS, native L1 (50 μg/ml), or L5 (50 μg/ml) for 24 h.

### CRBP1 cDNA transfection

For cell experiments of CRBP1 overexpression, the *crbp1* gene-transfected HK-2 cells were established. The pCMV6-GFP vector and human *crbp1* cDNA (gene number NM_002899) were purchased from OriGene Technologies Inc. (Rockville, MD). The CRBP1 cDNA was inserted into the Sgf 1/Mlu 1 site of the pCMV6-GFP expression vector plasmid (OriGene Technologies Inc.). HK-2 cells were transfected by using pCMV6-*crbp1*-I-GFP or pCMV6-GFP vector with Lipofectamine 2000 (Invitrogen). Cells were incubated in Opti-MEM (Invitrogen) at 37°C for 5 h and then placed in freshly changed culture medium for experiments. These cells were treated with PBS, native L1 (50 μg/ml), or L5 (50 μg/ml) for 24 h.

### Western blot

The protein of kidney, HK-2, or RTECs was extracted with M-PER mammalian protein extraction reagent (Pierce Biotechnology, Rockford, IL). The protein of samples was separated with SDS-PAGE. The separated proteins on SDS-PAGE were transferred onto PVDF membrane (Millipore) with electrophoresis. Then, the PVDF membrane was blocked with TBS with 0.2% Tween 20 (TBS-T) containing 5% skim milk at 4°C overnight. To detect LOX1, STRA6, CRBP1, RARα, RARγ, RXRα, pJNK, JNK, p-p38MAPK, p38MAPK, pSmad2, Smad2, TGFβ_1_, caspase 3, or collagen 1 protein expression, the PVDF membrane was incubated with diluted primary antibodies in TBS-T containing 5% skim milk. After washing the membrane with TBS-T, the PVDF membrane was incubated with a 1:10,000 dilution of HRP-conjugated secondary antibody in TBS-T containing 5% skim milk. Western blots were detected by ECL detection kit (Millipore) to induce the chemiluminescence signal, which was captured by a luminescence imaging system.

### RT-PCR analysis

Total RNA from the kidneys of saline-, L1-, or L5-injected mice, and L5-injected LOX1^−/−^ mice was extracted with Trizol reagent (Invitrogen). Total RNA was synthesized to first-strand cDNA by employing the AccessQuick RT-PCR system (Promega, Madison, WI). Then, the first-strand cDNA was processed to amplify cDNA fragments of STRA6, CRBP1, RARα, or RXRα mRNA with the PCR core kit (Invitrogen). The primers of mouse STRA6, CRBP1, RARα, and RXRα mRNA were purchased from Santa Cruz Biotechnology Inc. The cDNA fragments of PCR product were electrophoresed on agarose gels in a 100 V constant voltage field. Gels were photographed by using a gel 1000 UV documentation system and analyzed by densitometry. All mRNA levels were normalized by the corresponding actin mRNA level.

### Real-time quantitative PCR

Total RNA from cell lysate was extracted with Trizol (Invitrogen) and converted to first-strand cDNA with Super Script III cDNA synthesis kit (Invitrogen). The cDNA of STRA6, CRBP1, RARα, and RXRα mRNA was amplified and quantified by SYBR Green I quantitative (q)PCR master mix kit (OriGene Technologies, Inc.) and qPCR primer. Primers were as follows: STRA6, forward 5′-CTGCCTTGGGCTCCTGGTGC-3′ and reverse 5′-AGTCAGCCAGAAGGGCCACG-3′ CRBP1(h), forward 5′-TAGAGA­TGAGA­GTGGAAGGTGTGGT-3′ and reverse 5′-GGGG­TGGC­TG­GA­CAT­TTTTG-3′ CRBP1(m), forward5′-GCG­CG­CTC­GA­C­G­T­CA­AC-3′ and reverse 5′-ACGATCTCTTTGTCTGGCTTCAG-3′ RARα(h), forward 5′-GGTCGGCGAGTGAGGGT-3′ and reverse 5′-TGG­GC­AA­ATACACTACGAACAACAG-3′ RARα(m), forward 5′-CCAGCACCAGCTTCCAGTCA-3′ and reverse 5′-ACTGCTGCTCTGGG­TC­TCGAT-3′ RXRα(h), forward 5′-TACTTCAGAACGGGAATGACAAAC-3′ and reverse 5′-CCAGTGATGTAGGTAAATAAGA­TAGAGGG-3′ RXRα(m), forward 5′-AACCCCAGCTCACCA­AT­­GACC-3′ and reverse 5′-AACAGGACAATGGCTCGCAGG-3′. Data analysis was performed by Fold_change_ = 2^(ΔCt treatment-ΔCt control)^.

### Histochemistry

After deparaffinization and rehydration, kidney sections of saline-, L1-, or L5-injected mice, or L5-injected LOX1^−/−^ mice were placed in 0.01 M sodium citrate buffer (pH 6.0) and heated in a microwave oven for 2.5 min at 720 W. For Mason’s trichrome stain, sections were stained according to the protocol of the manufacturer (Sigma-Aldrich). For IHC, sections were washed in PBS and incubated with 1% BSA for 30 min to block nonspecific staining. Sections were drained and incubated for 3 h at room temperature in a humidity chamber with respective antibody for IHC, including anti-STRA6 antibody (ABGENT), anti-collagen 1 antibody (Santa Cruz Biotechnology Inc.), or anti-vitamin A antibody (MyBioSource, San Diego, CA) diluted with antibody diluent (Dako, Carpentaria, CA). After washing in PBS, endogenous peroxidase activity was blocked by incubation in 0.3% H_2_O_2_ in methanol for 20 min, followed by sequential 10 min incubations with biotinylated link antibody and peroxidase-labeled streptavidin (Dako). Staining was completed after incubation with 3,3’-diaminobenzidine substrate-chromogen solution (Dako), and then counterstained with hematoxylin. Images from similar regions of sections of kidney were captured by bright field microscopy at 400× microscopic magnification.

### Hydroxyproline analysis

Protein samples of kidneys of saline-, L1-, L5-injected mice, and L5-injected LOX1^−/−^ mice were hydrolyzed by 6 M HCl at 110°C for 18 h, and then centrifuged at 18,000 *g* for 2 min, and finally dried by freezing. Hydroxyproline of renal samples was analyzed with a hydroxyproline analysis kit (Sigma-Aldrich).

### Immunofluorescence

Cells were plated in eight-chamber glass slides. After treatment, cells were fixed in 4% paraformaldehyde. After PBS washing, blocking reagent (Dako) was used to block nonspecific background staining for 24 h. After PBS washing, cells were incubated with the anti-STRA6 antibody (Santa Cruz Biotechnology Inc.) at 4°C overnight. After PBS washing, TRITC- or FITC-conjugated secondary antibody (Lonza, Walkersville, MD) was added to cells. Cell membranes were counterstained with green fluorescence-membrane stain (Invitrogen) or nuclei stain, DAPI (Lonza). Under a fluorescence microscope (400×), the red fluorescence images of STRA6 on cell membranes were visualized.

### Terminal transferase-mediated deoxyuridine triphosphate nick end-labeling assay

Cells were plated in eight-chamber glass slides. After treatment of L1 (50 μg/ml) or L5 (50 μg/ml) for 24 h, cells were fixed in 4% paraformaldehyde and then analyzed by using an in situ apoptosis detection kit (R&D Systems, Minneapolis, MN) in accordance with the manual of the manufacturer. In the immunofluorescence image of HK-2 cells, deoxyuridine triphosphate nick ends were counterstained with FITC and nuclei were counterstained with DAPI. The incorporation of apoptotic cells (green fluorescence) was visualized under fluorescence microscope (400×). In the histochemistry image of RTECs, deoxyuridine triphosphate nick ends were counterstained with HRP-developed 3,3’-diaminobenzidine solution and cells were counterstained with hematoxylin. The incorporation of apoptotic cells (deep brown) was visualized under optical microscope (200×). To quantify the apoptotic cells, cells were counted for the percentage of positive cells with image analysis software in each well. All scoring was performed blinded to the calculator on coded slides.

### ELISA

#### Analysis of TGF-β_1_ ELISA.

Cells were grown in plates for experiments. After treatment, culture medium of cells was collected to measure TGFβ_1_ concentrations with TGFβ_1_ Emax® ImmunoAssay Systems (Promega) for HK-2 cells or LEGEND MAX™ Total TGF-β_1_ ELISA kit (BioLegend, San Diego, CA) for RTECs.

#### Analysis of vitamin A and RA ELISA.

Kidneys of saline-, L1-, and L5-injected mice, and L5-injected LOX1^−/−^mice were homogenized and processed with tissue lysis buffer (Promega), and then lysates of kidney were collected to measure vitamin A or RA concentrations with ELISA kits. HK-2 and RETC cell experiments were collected and processed with cell lysis buffer (Promega), and then cell lysates were collected to measure concentrations of vitamin A or RA with ELISA kits. The vitamin A and RA ELISA kits were both purchased from MyBioSource.

### Statistical analysis

GraphPad Prism software (GraphPad Software, Inc., San Diego, CA) was used for statistical analysis. Data are expressed as the mean ± SE. Differences in measured variables between experimental and control groups were assessed by one-way ANOVA with Bonferroni test. Probability values of *P* < 0.05 were considered significant.

## RESULTS

### L5 injection suppresses STRA6/CRBP1/RARs/RXRα, increases pJNK and p-p38, and causes renal injury via LOX1 in vivo

**Fig. 1.**
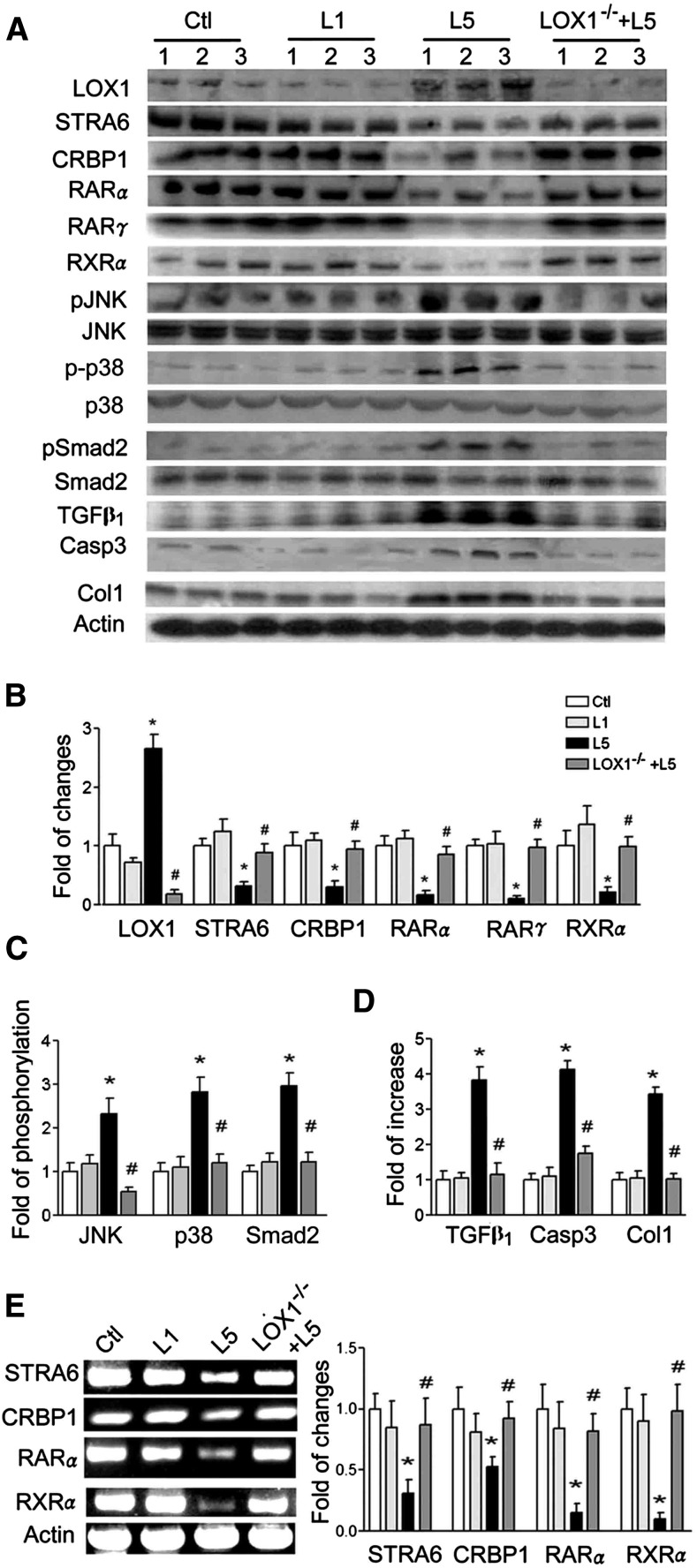
L5 decreases STRA6 cascades and injures the kidney. The protein of kidney samples (n = 3) was extracted from saline-injected (Ctl), L1-injected (L1), and L5-injected (L5) mice, as well as L5-injected LOX1^−/−^ mice (LOX1^−/−^+L5) after injection for 4 weeks. A: Western blots showed that L5 reduced STRA6, CRBP1, RARα, RARγ, and RXRα, but increased LOX1, pJNK, p-p38MAPK (p-p38), TGFβ_1_, pSmad2, caspase 3 (Casp3), and collagen 1 (Col1) in L5-injected mice. In LOX1^−/−^ mice, these changes caused by L5 were diminished. B: Bar graphs show that LOX1 increased and STRA6, CRBP1, RARα, RARγ, and RXRα decreased in L5-injected mice. These changes caused by L5 were attenuated in L5-injected LOX1^−/−^ mice. C: Bar graphs indicate that pJNK/JNK, p-p38/p38, and pSmad2/Smad2 ratios increased in L5-injected mice. These changes caused by L5 were attenuated in L5-injected LOX1^−/−^ mice. D: TGFβ_1_, caspase 3, and collagen 1 levels of L5-injected mice increased. These changes caused by L5 were recovered in L5-injected LOX1^−/−^ mice. E: RT-PCR analysis shows that L5 decreased PCR products of STRA6, CRBP1, RARα, and RXRα in L5-injected mice, but there was no effect in L5-injected LOX1^−/−^ mice. All results are represented as mean ± SE; *P < 0.05 versus Ctl and L1; ^#^*P* < 0.05 versus L5.

L5 injection remarkably increased LOX1 expression, but suppressed STRA6, CRBP1, RARα, RARγ, and RXRα expression compared with saline and L1 injection in kidneys of C57B6/J mice (**Fig. 1A, B**). The pJNK p-p38 and pSmad2 increased in kidneys of L5-injected mice as compared with other groups (Fig. 1A, C). TGFβ_1_, caspase 3, and collagen 1 levels were higher in kidneys of L5-injected mice than saline-injected and L1-injected mice (Fig. 1A, D). All these changes of LOX1, STRA6, CRBP1, RARα, RARγ, RXRα, pJNK/JNK, p-p38/p38, pSmad/Smad2, TGFβ_1_, caspase 3, and collagen 1 values in the kidneys of L5-injected mice were reversed in L5-injected LOX1^−/−^ mice (Fig. 1A–D). Similarly, mRNA levels of STRA6, CRBP1, RARα, and RXRα (Fig. 1E) were suppressed in the kidneys of L5-injected mice. These changes were reversed in L5-injected LOX1^−/−^ mice (Fig. 1E).

### L5 decreases STRA6-immunoreactive density but increases collagen 1-immunoreactive density and trichrome stain of kidney in vivo

**Fig. 2.**
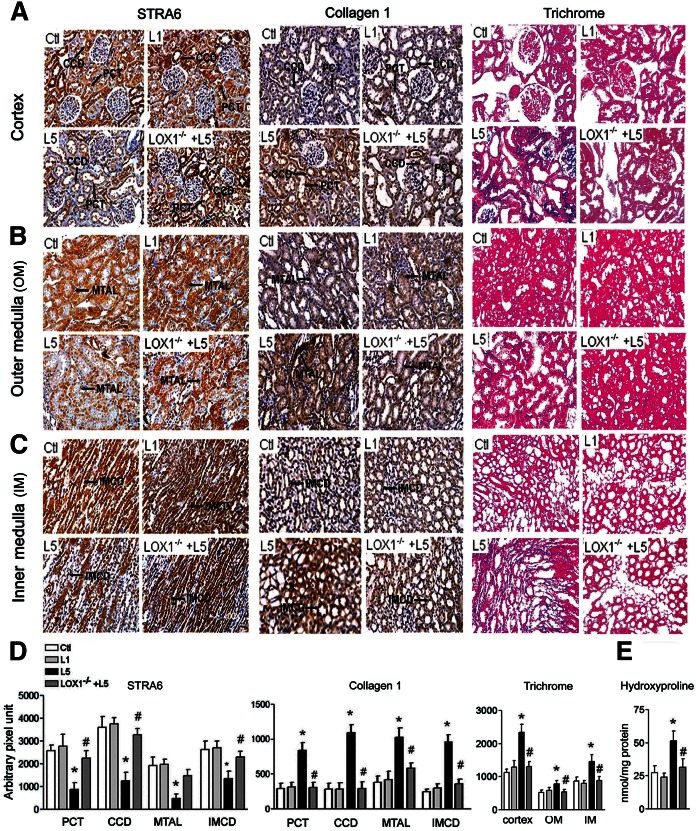
L5 decreases STRA6, but increases collagen 1, immunohistochemical staining, trichrome staining, and hydroxyproline concentration in the kidneys. Representative images show immunoreactive staining for STRA6, collagen 1, and Mason’s trichrome stain in cortex (A), outer medulla (B), and inner medulla (C) from saline-injected (Ctl), L1-injected (L1), L5-injected (L5), and L5-injected LOX1^−/−^ mice (LOX1^−/−^+L5). D: Quantitative analysis showed that STRA6 immunostaining intensity increased but collagen 1 intensity decreased in the PCT, CCD, MTAL, and IMCD in L5-injected mice, as compared with Ctl and L1 mice or L5-injected LOX1^−/−^ mice. Intensity of trichrome stain increased in the cortex, outer medulla (OM), and inner medulla (IM) of L5-injected mice. E: Bar graph shows that hydroxyproline concentration increased in the kidneys of L5-injected mice. All results are represented as mean ± SE. *P < 0.05 versus Ctl and L1; ^#^*P* < 0.05 versus L5.

**Figure 2A–C** shows the immunohistochemical intensity for STRA6, collagen 1, and trichrome stain in the renal cortex (Fig. 2A), outer medulla (Fig. 2B), and inner medulla (Fig. 2C) of one representative of four groups. A considerable decrease of immunostaining for STRA6 in the proximal convoluted tubule (PCT), cortical collecting duct (CCD), medullary thick ascending limb (MTAL), and inner medullary collecting duct (IMCD) was found in L5-injected mice as compared with saline-injected, L1-injected, and L5-injected LOX1^−/−^ mice. In contrast, the staining for collagen 1 was considerably enhanced in the PCT, CCD, MTAL, and IMCD in L5-injected mice as compared with saline-injected, L1-injected, and L5-injected LOX1^−/−^ mice. Trichrome stain (blue) of renal cortex (Fig. 2A), outer medulla (Fig. 2B), and inner medulla (Fig. 2C) was enhanced in L5-injected mice, but not in L1-injected or L5-injected LOX1^−/−^ mice. The bar graphs for quantification of STRA6 and collagen 1 immunostaining density in the PCT, CCD, MTAL, and IMCD are presented in Fig. 2D. The bar graphs for quantifying intensity of trichrome stain (blue) in renal cortex, outer medulla, and inner medulla are presented in Fig. 2D. Figure 2E shows that L5-injection significantly increased hydroxyproline in the kidneys of L5-injected C57B6/J mice, but not LOX1^−/−^ mice.

### L5 suppresses STRA6/CRBP1/RAR and increases LOX1 and pJNK, and causes renal injury via LOX1 in HK-2 cells

**Fig. 3.**
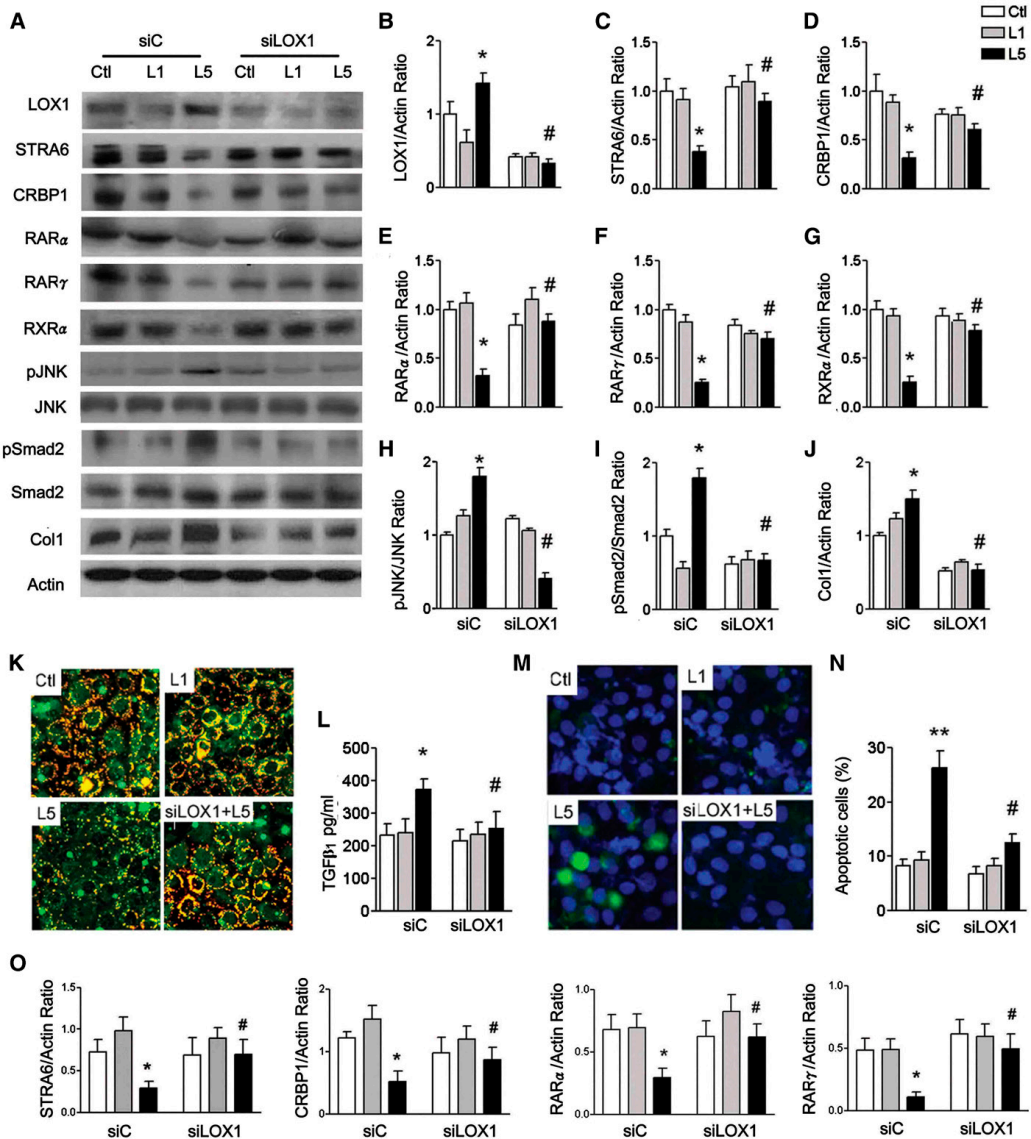
L5 suppresses STRA6 cascades and increases cell injury in HK-2 cells. A: Western blots show LOX1, STRA6, CRBP1, RARα, RARγ, RXRα, pJNK, pSmad2, and collagen 1 (Col1) in control siRNA (siC)-transfected or LOX1 siRNA (siLOX1)-transfected HK-2 cells (n = 3) after PBS (Ctl), L1, or L5 treatment for 24 h. In L5-treated HK-2 cells, quantitative analysis showed that LOX1 (B) protein levels significantly increased; STRA6 (C), CRBP1 (D), RARα (E), RARγ (F), and RXRα (G) protein levels decreased; pJNK/JNK (H) and pSmad2/Smad2 (I) ratios increased; and collagen 1 (J) increased. These changes were reversed in LOX1 siRNA-transfected L5-injected HK-2 cells, as compared with L5-injected siC mice. K: Representative images (400×) show immunoreactive staining for STRA6 (red fluorescence) emerged to cell membrane staining (green fluorescence) on chamber slides of Ctl-, L1-, and L5-treated cells, and L5-treated LOX1 siRNA-transfected cells. L: ELISA showed an increase of TGFβ_1_ concentration in L5-treated siC cells, but not in L5-treated siLOX1 cells. M: Cytochemistry images show the nucleus of apoptotic cells (green fluorescence) emerged to staining of nucleus (blue fluorescence) in Ctl-, L1-, and L5-treated cells, and in L5-treated LOX1 siRNA-transfected cells. N: Number of apoptotic cells significantly increased in L5-treated siC cells, but not in L5-treated siLOX1 cells. O: Real-time PCR analysis showed that LOX1 siRNA significantly increased mRNA levels of STRA6, CRBP1, RARα, and RXRα in L5-treated HK-2 cells. All results are represented as mean ± SE. *P < 0.05 versus Ctl- and L1-siC; ^#^*P* < 0.05 versus L5-siC group.

To further verify these results observed in vivo, we added native L1 and L5 to human RTECs (HK-2) with control siRNA- or LOX1 siRNA-transfected HK-2 cells for 24 h. L5 treatment strongly enhanced LOX1 in HK-2 cells (**Fig. 3A, B**). L5 treatment decreased STRA6, CRBP1, RARα, RARγ, and RXRα values, but increased pJNK, pSmad2, and collagen 1 in HK-2 cells (Fig. 3C–J). These changes caused by L5 treatment were attenuated in LOX1 siRNA-transfected HK-2 cells (Fig. 3C–J). Immunofluorescence staining for STRA6 (orange) was weaker in L5-treated HK-2 cells compared with control and L1-treated cells. LOX1 siRNA reversed the decrement of STRA6 in L5-treated HK-2 cells (Fig. 3K). Additionally, L5 treatment significantly increased TGFβ_1_ concentration, measured by ELISA, in culture medium of L5-treated HK-2 cells compared with control and L1-treated cells. L5-induced increase of TGFβ_1_ concentration was attenuated by LOX1 siRNA (Fig. 3L). Using terminal transferase-mediated deoxyuridine triphosphate nick end-labeling assay, L5 treatment significantly increased the number of apoptotic cells (green) in L5-treated HK-2 cells. In LOX1 siRNA HK-2 cells, apoptotic cells were not different from control and L1-treated cells (Fig. 3M, N). In transcription level, STRA6, CRBP1, RARα and RARγ mRNA levels were reduced by L5 treatment in HK-2 cells (Fig. 3O). However, LOX1 siRNA recovered these gene expressions caused by L5 in HK-2 cells (Fig. 3O).

### JNK and p38MAPK inhibitors reverse L5-induced suppression of STRA6 cascades and renal injury in RTECs

**Fig. 4.**
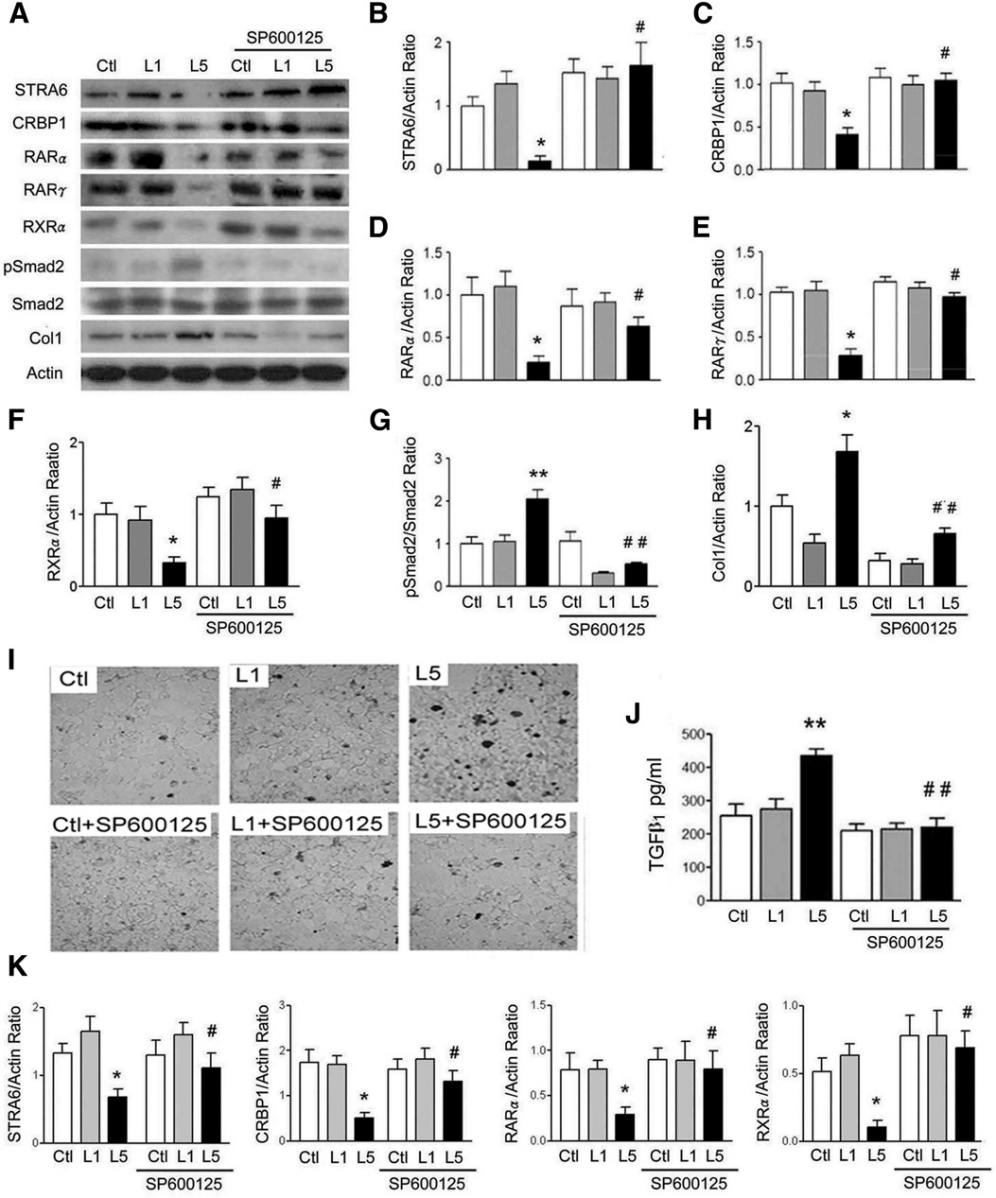
JNK inhibitor represses L5 effect on STRA6 cascades and renal injury. A: Western blots show STRA6, CRBP1, RARα, RARγ, RXRα, pSmad2, and collagen 1 (Col1) of Ctl, L1-, or L5-treated RETCs with or without SP600125 treatment for 24 h (n = 3). Quantitative analysis showed that L5 treatment decreased STRA6 (B), CRBP1 (C), RARα (D), RARγ (E), and RXRα (F), but increased the pSmad2/Smad2 ratio (G) and collagen 1 (H). All of these effects of L5 were reversed in SP600125-treated RETCs under L5-stimulation. I: Representative optical microscopy images show nuclei of apoptotic cells (thick dark stain) in Ctl, L1-treated, and L5-treated cells with or without SP600125 treatment. SP600125 decreased the nuclei of apoptotic cells in RETCs under L5-stimulation. J: ELISA showed that SP600125 treatment attenuated the increase of TGFβ_1_ concentration in the culture medium of L5-treated RETCs. K: Real-time PCR analysis revealed that SP600125 significantly recovered the decreased mRNA levels of STRA6, CRBP1, RARα, and RXRα in L5-treated RETCs. All results are represented as mean ± SE. *P < 0.05, **P < 0.01 versus Ctl and L1; ^#^*P* < 0.05, ^##^*P* < 0.05 versus L5.

L5-induced suppression of STRA6, CRBP1, RARα, RARγ, and RXRα, as well as L5-induced increases of pSmad2/Smad2 and collagen 1, were reversed by JNK inhibitor, SP600125 (**Fig. 4A–H**) and also by p38MAPK inhibitor, SB203580 (supplemental Fig. S1A–H) in L5-treated RTECs. In optical microscopy images of terminal transferase-mediated deoxyuridine triphosphate nick end-labeling analysis, L5-induced increase of apoptotic cell nuclei (thick dark stain) was recovered by SP600125 and by SB203580 (Fig. 4I, supplemental Fig. S1I). Higher TGFβ_1_ concentration in cultured medium of L5-treated RTECs was reversed by the addition of SP600125 (Fig. 4J) and SB203580 (supplemental Fig. S1J). In transcription level, decreases of STRA6, CRBP1, RARα, and RXRα mRNA were also reversed by SP600125 (Fig. 4K).

### CRBP1 transfection reverses the suppression of STRA6 cascades and cell damage in L5-treated renal epithelial cells

**Fig. 5.**
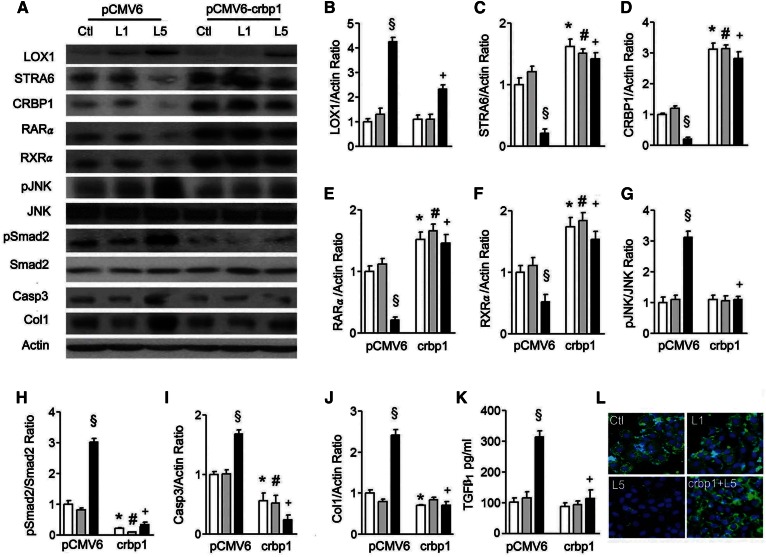
The crbp1 gene transfection reverses L5 effects on STRA6 cascades and renal cell injury. A: Western blots show LOX1, STRA6, CRBP1, RARα, RXRα, pJNK, pSmad2, and collagen 1 (Col1) expression in cell lysate of pCMV6-transfected and pCMV6-crbp1-transfected HK-2 cells under PBS (Ctl), L1, or L5 treatment for 24 h (n = 3). Quantitative analysis showed a significant difference for the increase of LOX1/actin (B); the decrease of STRA6/actin (C), CRBP1/actin (D), RARα/actin (E), and RXRα/actin (F); the increase of pJNK/JNK (G), pSmad2/Smad2 (H), caspase 3/actin (I), and collagen 1/actin (J) under L5 stimulation in pCMV6-transfected cells. These changes caused by L5 treatment were reversed in pCMV6-crbp1-transfected cells. K: ELISA showed that pCMV6-crbp1-transfection reversed the elevation of TGFβ_1_ concentration under L5 treatment in the culture medium of HK-2 cells. L: Cytochemistry images show immunoreactive staining of STRA6 (green fluorescence) was recovered by pCMV6-crbp1-transfection in L5-treated HK-2 cells (crbp1+L5). All results are represented as mean ± SE. ^§^*P* < 0.05 versus Ctl- and L1-treated pCMV6 groups; *P < 0.05 versus Ctl-treated pCMV6 group; ^#^*P* < 0.05 versus L1-treated pCMV6 group; ^+^*P* < 0.05 versus L5-treated pCMV6 group.

Because STRA6-mediated retinol transport requires the presence of CRBP1, and we established stable crbp1 gene-transfected HK-2 cells to investigate whether *crbp1* transfection could affect STRA6 cascades in cells cultured with native L1 and L5. We found that *crbp1* gene transfection could significantly reverse L5 treatment-induced increase of LOX1, L5-induced suppression of STRA6, CRBP1, RARα, and RXRα, or L5-induced increases of pJNK, pSmad2, caspase 3, and collagen 1 expression in L5-stimulated HK-2 cells (**Fig. 5A–J**). Elevated TGFβ_1_ concentration in cultured medium of L5-treated HK-2 cells was also suppressed by *crbp1* transfection (Fig. 5K). The decrease of STRA6 immunofluorescence staining (green) was also recovered by *crbp1* transfection in L5-cultured HK-2 cells (Fig. 5L).

### Retinoid concentration is reduced by L5 treatment in vivo and in vitro

**Fig. 6.**
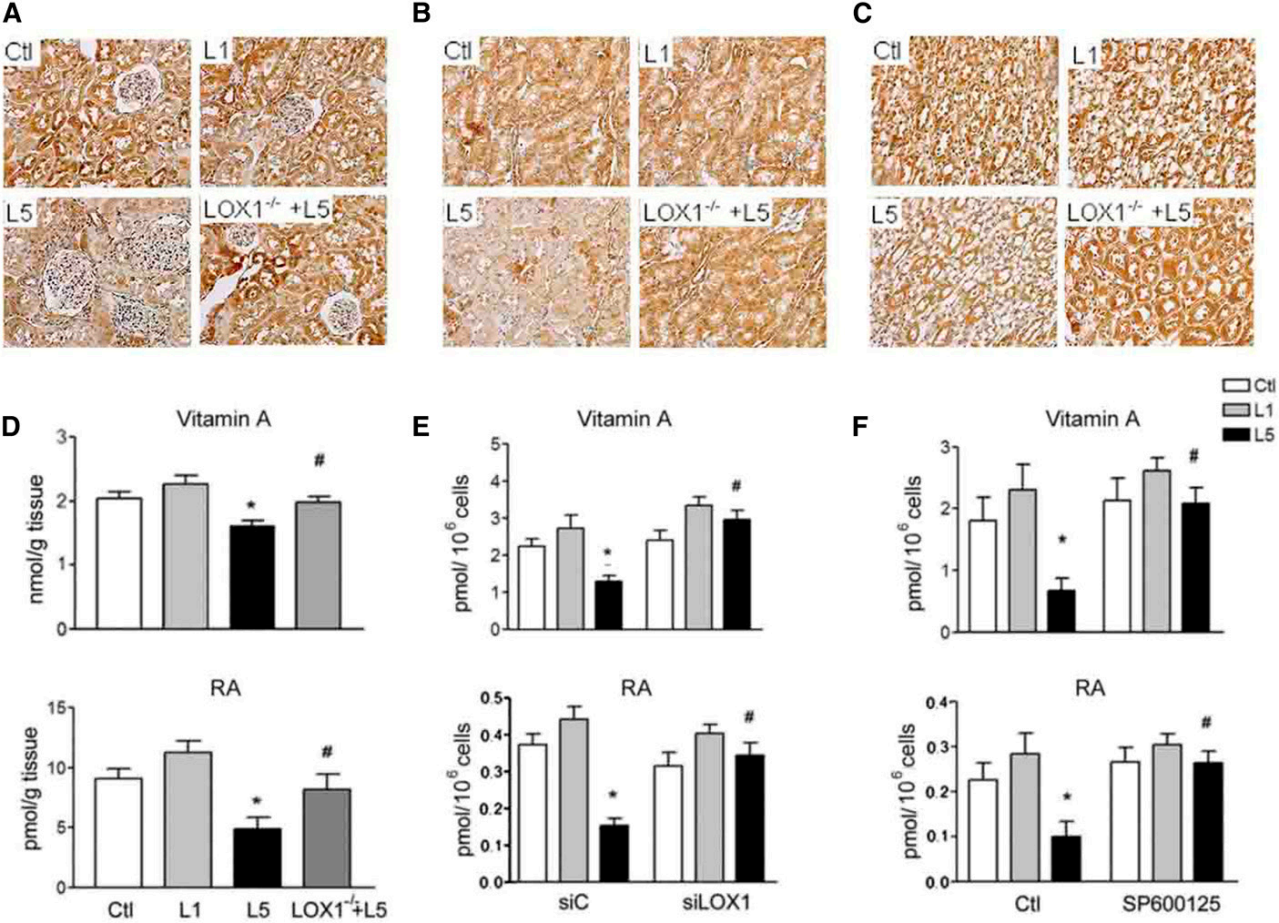
L5 decreases vitamin A and RA in kidneys and renal cells. Representative images show immunohistochemical staining for vitamin A in sections of cortex (A), outer medulla (B), and inner medulla (C) from saline-injected (Ctl), L1-injected (L1), L5-injected (L5), and L5-injected LOX1^−/−^ mice (LOX1^−/−^+L5). D: Vitamin A and RA concentration decreased in the kidneys of L5-injected mice as compared with Ctl, L1, and L5 LOX1^−/−^ mice (n = 3). Error bars of all experiments indicate mean ± SE. *P < 0.05 versus Ctl and L1; ^#^*P* < 0.05 versus L5. E: ELISA assay showed that LOX1 siRNA transfection reversed the reduction of vitamin A and RA level under L5 stimulation in HK-2 cells (n = 3). Error bars of all experiments indicate mean ± SE. *P < 0.05 versus Ctl and L1-treated siC cells; ^#^*P* < 0.05 versus L5-treated siC cells. F: ELISA revealed that SP600125 treatment reversed the reduction of vitamin A and RA level under L5 stimulation in cell lysate of RETCs (n = 3). All results are represented as mean ± SE. *P < 0.05 versus Ctl and L1; ^#^*P* < 0.05 versus L5.

Immunohistochemistry by using anti-vitamin A antibody showed a considerable decrease of vitamin A staining density in the renal cortex (**Fig. 6A**), outer medulla (Fig. 6B), and inner medulla (Fig. 6C) of L5-injected mice in comparison with saline-injected, L1-injected, and L5-injected LOX1^−/−^ mice. Vitamin A and RA concentrations were diminished in the kidneys of L5-injected mice, as compared with saline-injected, L1-injected, and L5-injected LOX1^−/−^ mice (Fig. 6D). LOX1 siRNA transfection reversed the L5-induced decrease of vitamin A and RA concentrations in renal cells (Fig. 6E). SP600125 (Fig. 6F) and SB203580 (supplemental Fig. S1K) could recover the reduction of vitamin A and RA concentration in L5-treated RTECs.

### STRA6 RNA silence decreases STRA6 cascades and causes cell damage

**Fig. 7.**
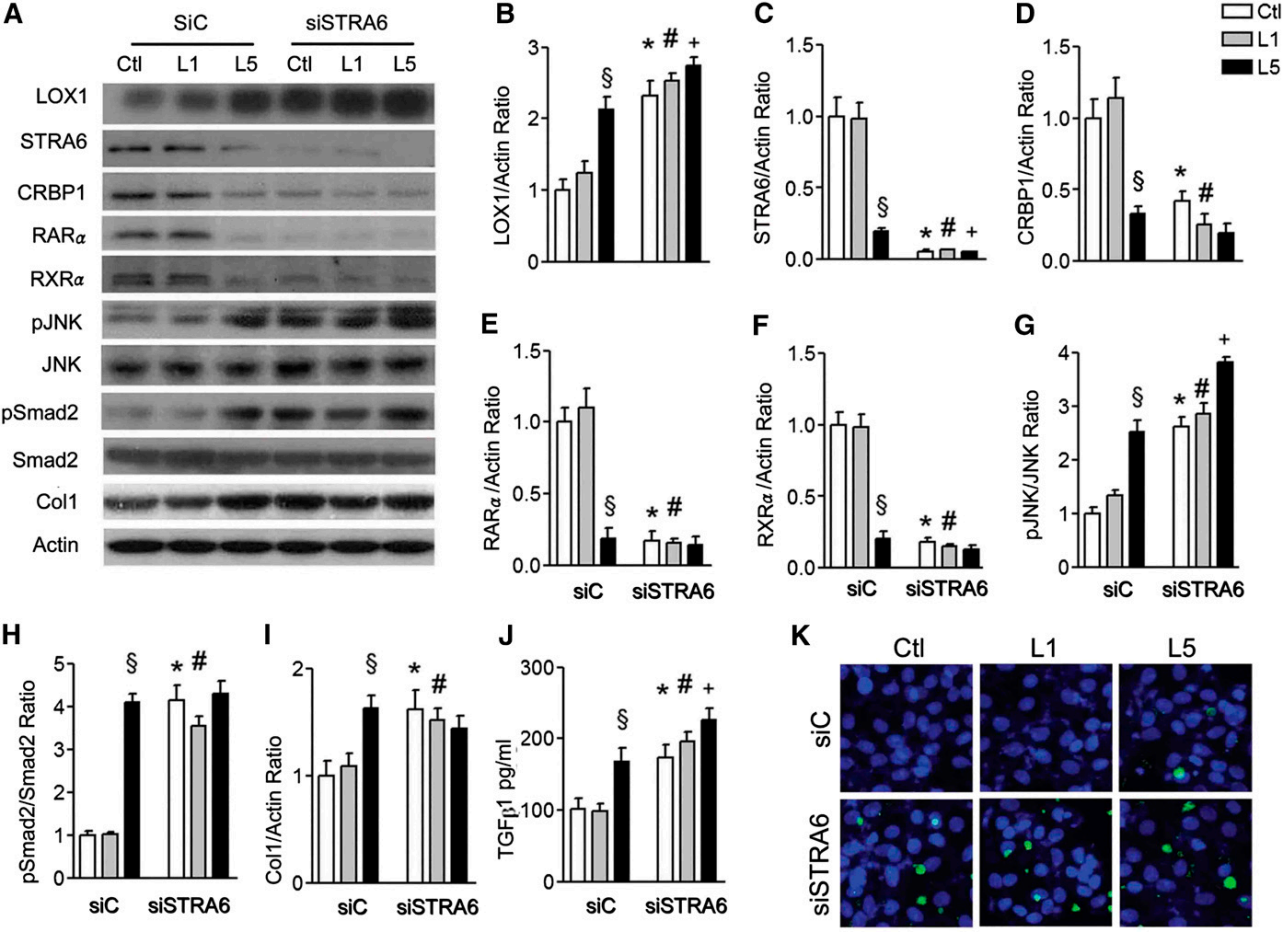
STRA6 siRNA damages STRA6 cascades and induces renal cell injury. A: Western blots show LOX1, STRA6, CRBP1, RARα, RXRα, pJNK, pSmad2, and collagen 1 (Col1) in cell lysate of STRA6 siRNA (siSTRA6) or control siRNA (siC) transfected HK-2 cells cultured in PBS (Ctl)-, L1-, and L5-treated HK-2 cells for 24 h (n = 3). Quantitative analysis showed significance for the increase of LOX1/actin (B); the decrease of STRA6/actin (C), CRBP1/actin (D), RARα/actin (E), RXRα/actin (F), pJNK/JNK (G), pSmad2/Smad2 (H), and collagen 1/actin (I) in STRA6 siRNA-transfected HK-2 cells in Ctl and L1 groups. J: ELISA also showed a significant increase of TGFβ_1_ in culture medium of siSTRA6-transfected HK-2 cells in Ctl, L1, and L5 groups. K: Cytochemistry images show that STRA6 siRNA transfection increased the nuclei of apoptotic cells (green fluorescence) in Ctl- and L1-treated cells. All results are represented as mean ± SE. ^§^*P* < 0.05 versus and L1-treated siC cells; *P < 0.05 versus Ctl-treated siC cells; ^#^*P* < 0.05 versus L1-treated siC cells; ^+^*P* < 0.05 versus L5-treated siC cells.

To investigate whether suppression of STRA6 cascades was involved in kidney damage, we silenced STRA6 expression in HK-2 cells with siRNA. The efficiency of dose-dependent STRA6 siRNA was adapted to significantly block STRA6 mRNA and protein expression in HK-2 cells. In this experiment, STRA6 siRNA transfection significantly increased LOX1, suppressed CRBP1 and RARα, but increased pJNK/JNK, pSmad2/Smad2, and collagen 1 expression in PBS-treated and L1-treated HK-2 cells (**Fig. 7A, C–I**). It markedly increased TGFβ_1_ concentration in cultured medium of PBS-treated and L1-treated HK-2 cells (Fig. 7J). Furthermore, silencing stra6 also increased apoptotic cells (green fluorescence) in PBS-treated and L1-treated HK-2 cells (Fig. 7K).

## DISCUSSION

**Fig. 8.**
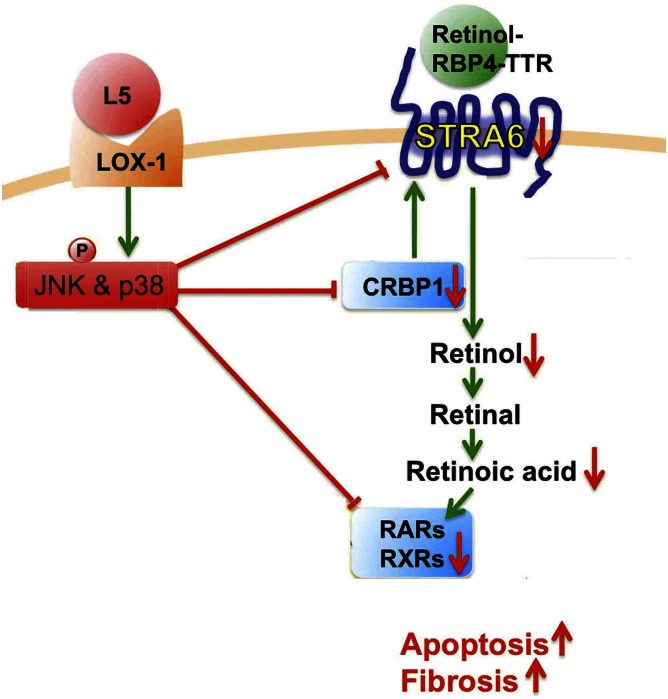
The schematic mechanism of L5 on STRA6 cascade suppression and kidney injury. L5-activated LOX1/JNK and p38MAPK pathway suppresses STRA6/CRBP1/retinol/RA/RARs/RXRs cascade and, thereafter, contributes to apoptosis and fibrosis of the kidney.

In the current study, we indicate that L5 treatment can cause renal apoptosis and fibrosis via LOX1 in L5-injected mice and L5-treated HK-2 cells, while L1 treatment cannot. Especially, this study first reveals the mechanism that L5 simultaneously suppresses the STRA6/CRBP1/retinol/RA/RARs/RXRα cascade via activating the LOX1/JNK pathway (**Fig. 8**). Particularly, we furthermore show that the suppression of STRA6 cascades is involved in renal apoptosis and fibrosis caused by L5 treatment.

oxLDL has been commonly utilized to provide direct evidence for the causal effect of dyslipoproteinemia-related kidney damage. However, oxLDL was artificially produced by oxLDL in the presence of CuSO_4_ in most investigations (9–14). Here we showed that native L5 injection could markedly increase caspase 3, collagen 1 in kidneys of C57B6/J mice, but not in LOX1^−/−^ mice. Additionally, L5 treatment also increased these proteins and apoptotic cells in HK-2 cells, but not in LOX1 siRNA HK-2 cells. The immunostaining intensity of collagen 1 and trichrome in the kidneys of L5-injected mice was strongly expressed. Altogether, this study provides new evidence that dyslipoproteinemia can cause renal apoptosis and fibrosis by utilizing native L5.

L5 is not recognized by the normal LDL receptor, but is internalized by LOX1. LOX1 is one of several receptors for oxLDL, and its activation can result in oxLDL-induced endothelial dysfunction (37, 38). In our previous studies, L5 induced endothelial cell apoptosis and platelet aggregation in a LOX1-dependent manner (16, 17). LOX1 overexpression is also reported in renal tubular cells and capillaries in the kidneys of rats with dyslipidemia and diabetes (9, 10). Intravenous injection of LOX-1 antibody could prevent the renal injury in these rats (10). Consistently, this study furthermore shows overexpression of LOX1 in kidneys of native L5-injected mice and L5-treated HK-2 cells. Additionally, LOX1 gene knockdown and LOX1 RNA silencing can attenuate renal apoptosis and fibrosis induced by L5 in mice and renal cells. These results elucidate that native L5 causes kidney damage via LOX1.

Although RA and RARα agonist have been demonstrated to provide protection in several experimental models of kidney disease (31–35), there are very few studies to report the change of STRA6/CRBP1 cascades in kidney diseases. In mice with Adriamycin nephropathy, it was demonstrated that albumin prevented podocyte differentiation from human renal progenitors in vitro by sequestering RA (34). A great reduction in RA and RARβ levels was reported in the glomeruli of human immunodeficiency-associated kidney disease (35). Reduced retinol dehydrogenase 1 and 9 levels were also observed in human immunodeficiency transgenic mice (35, 36). In this study, retinol and RA values in the kidneys of L5-injected mice and in L5-treated renal cells considerably decreased in comparison with L1-treated mice and renal cells. To our knowledge, this study is the first to indicate that native L5 can remarkably reduce STRA6, CRBP1, retinol, RA, RARα, and RXRα expression in kidneys of mice and in renal cells. LOX1 knockdown and LOX1 RNA silencing reversed these alterations. Additionally, immunostaining intensity of STRA6 was markedly repressed, while collagen 1 immunostaining and trichrome stain were reciprocally increased by L5 injection in renal tubules and collect ducts of L5-injected mice. These changes were attenuated in L5-injected LOX1^−/−^ mice. Therefore, these data reveal that retinoid homeostasis in the kidney is suppressed by L5 treatment via repressing STRA6/CRBP1/retinol/RA/RARs signaling in the pathological process of kidney disease.

STRA6-mediated retinol transport into cells requires CRBP1, an intracellular retinol acceptor (25, 29). To investigate whether the suppression of STRA6 and CRBP1 can activate fibrotic process and apoptosis, we performed *crbp1* gene transfection and STRA6 siRNA transfection. Notably, transfection of the *crbp1* gene could significantly reverse increases of fibrotic and apoptotic proteins, while it concurrently reversed the suppression of STRA6/RARα/RXRα in L5-stimulated HK-2 cells. Conversely, STRA6 siRNA transfection significantly increased pJNK, pSmad2, collagen 1, and TGFβ_1_ levels and apoptotic cells, whereas it negatively regulated CRBP1, RARα, and RXRα in PBS-treated and L1-treated HK-2 cells. These results reveal that L5-mediated suppression of STRA6/CRBP1/retinol/RA/RARs/RXR cascades participates in L5-mediated kidney apoptosis and fibrosis.

Some studies propose that the high glucose-activated JNK pathway could suppress the expression of RAR and RXR and then significantly contribute to high glucose-induced cardiomyocyte apoptosis (39, 40). In this study, JNK and p38MAPK phosphorylation was activated in the kidneys of L5-injected mice and L5-treated renal cells, but was not activated in LOX1 knockdown models in vivo and vitro. JNK and p38MAPK inhibitors can reverse the suppression of the STRA6/CRBP1/retinol/RA/RAR/RXR cascade, as well as the induction of renal apoptosis and fibrosis. These results indicate that JNK and p38MAPK signaling is activated by L5 to process kidney apoptosis and fibrosis after the activation of LOX1.

In summary, we demonstrate that electronegative L5 can cause kidney apoptosis and fibrosis and simultaneously suppress STRA6, CRBP1, retinol, RA, RARs, and RXRα. The alteration of STRA6 cascades is involved in kidney apoptosis and fibrosis caused by L5 treatment. These findings implicate that the suppression of STRA6 signaling may participate in dyslipidemia-mediated kidney disease.

## Supplementary Material

Supplemental Data
