# Supplementary material for: Electronegative low density lipoprotein induces renal apoptosis and fibrosis: STRA6 signaling involved
Source: J Lipid Res. 2016 Aug;57(8):1435–46. doi: 10.1194/jlr.M067215 (PMC4959859; doi:10.1194/jlr.M067215)
Supplement: Supplemental Data [file supp_57_8_1435__index.html]

Electronegative Low-Density Lipoprotein Induces Renal Apoptosis and Fibrosis: STRA6 Signaling Involved — Electronegative low density lipoprotein induces renal apoptosis and fibrosis: STRA6 signaling involved — Supplemental Data 

# Electronegative low density lipoprotein induces renal apoptosis and fibrosis: STRA6 signaling involved

## Supplemental Data

- Fig. S1 (.pdf, 2.9 MB) - Supplemental Figure 1
