# Supplementary material for: Electronegative low density lipoprotein induces renal apoptosis and fibrosis: STRA6 signaling involved
Source: J Lipid Res. 2016 Aug;57(8):1435–46. doi: 10.1194/jlr.M067215 (PMC4959859; doi:10.1194/jlr.M067215)
Supplement: Supplemental Data [file 10.1194_M067215_jlr.M067215-1.pdf]

SUPPLEMENT

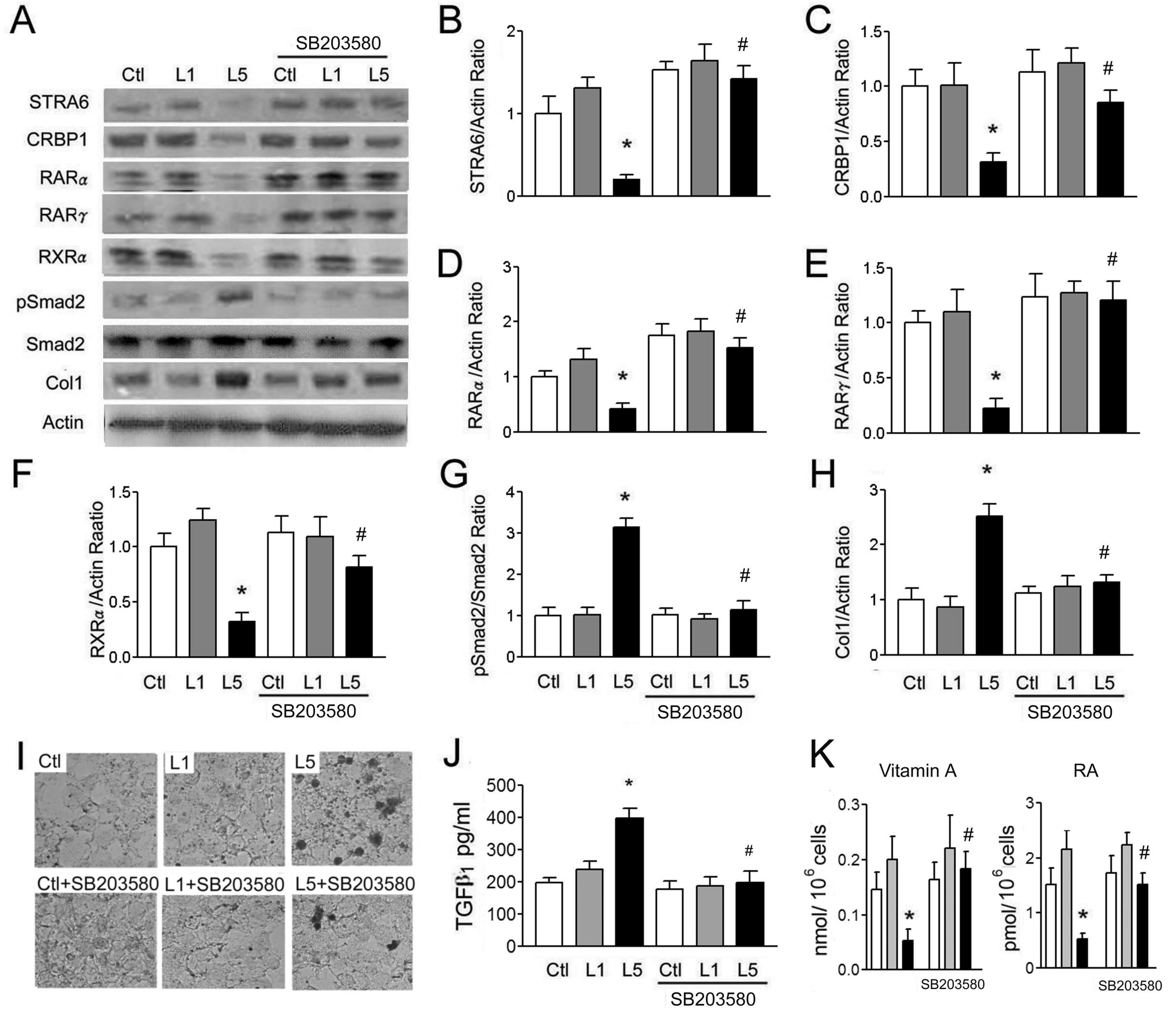

Figure S1. p38MAPK inhibitor represses L5 effect on STRA6 cascades and renal injury. (A) Western blots showed STRA6, CRBP1, RARα, RARγ, RXRα, pSmad2, and collagen 1 (Col1) of Ctl, L1-, or L5-treated RETC cells with or without SB203580 treatment for 24 hours (n=3). Quantitative analysis showed that L5 treatment decreased STRA6 (B), CRBP1(C), RARα (D), RARγ (E), and RXRα (F), but increased pSmad2/Smad2 ratio (G) and Collagen 1 (H). All these effects of L5 were reversed in SB203580-treated RETC cells under L5-stimulation. (I) Representative optical microscopy images showed nuclei of apoptotic cells (thick dark stain) in Ctl, L1-treated, L5-treated cells with or without SB203580 treatment. SB203580 decreased nuclei of apoptotic cells in RETC cells under L5-stimulation. (J) ELISA showed SB203580 treatment attenuated the increase of $TGF\beta_1$ concentration in culture medium of L5-treated RETC cells. (K) ELISA revealed that SB204580 treatment reversed the reduction of vitamin A and RA level under L5 stimulation in of RETC cells. * $P<0.05$ vs. Ctl and L1; $^{\#}P<0.05$ vs. L5.
